# Contextualising implementation interventions for promoting outpatient integrative Chinese–western oncology service delivery and utilisation in Hong Kong: a Delphi study

**DOI:** 10.1186/s12906-025-04779-0

**Published:** 2025-03-28

**Authors:** Leonard Ho, Ming Hong Kwong, Angus S. C. Li, Fai Fai Ho, Claire C. W. Zhong, Charlene H. L. Wong, Vincent C. H. Chung

**Affiliations:** 1 Jockey Club School of Public Health and Primary Care, Faculty of Medicine, Chinese University of Hong Kong, Shatin, Hong Kong; 2 School of Chinese Medicine, Faculty of Medicine, Chinese University of Hong Kong, Shatin, Hong Kong

**Keywords:** Cancer, Integrative medicine, Implementation science, Delivery of health care, Qualitative research

## Abstract

**Introduction:**

The rapidly rising incidence and prevalence of cancer pose a financial burden on Hong Kong’s health system. This study aimed to co-create an outpatient integrative oncology (IO) service delivery model for the territory that bridges the District Health Centres (DHCs; local coordinators for medical and social service providers), private traditional Chinese medicine practitioners (TCMPs), and private oncologists and to establish stakeholder-recommended implementation interventions (IIs) for promoting service delivery and utilisation.

**Methods:**

We conducted individual semi-structured contextual interviews to develop a preferred outpatient model that would enable DHC-based IO interprofessional collaborations and to refine relevant IIs developed for facilitating the implementation of the model. Then, we conducted a Delphi survey to contextualise and finalise the IIs using the APEASE criteria.

**Results:**

After interviewing 11 local stakeholders, a model was proposed that IO specialist nurses in DHCs would coordinate referrals between private TCMPs and oncologists and evaluate service delivery. Thirty-six IIs were refined to support model implementation. This model presented the possible role of cancer nurse specialists in leading and coordinating interprofessional cancer care between traditional and conventional medicine. In the Delphi study, 21 local stakeholders achieved positive consensus on 35 IIs (agreement 76.2–100%). Affordability was the most critical criterion in determining the recommendation of IIs.

**Conclusions:**

Along with the 35 local stakeholder-recommended IIs, our proposed nurse-led model provided insights into forging the partnership between the nurse specialists, private TCMPs, and oncologists to provide outpatient IO services. Further research is expected to seek opinions from policymakers regarding the potential administrative implementation determinants.

**Supplementary Information:**

The online version contains supplementary material available at 10.1186/s12906-025-04779-0.

## Introduction

### International trend of providing oncology services in outpatient settings

The incidence and prevalence of cancer are rising rapidly, and it is anticipated that there will be 28 million new cases of cancer each year by 2040 [1]. While countries are endeavouring to mitigate the financial impact incurred by providing oncology services, researchers have been proposing different outpatient service delivery models for routine oncology services, including palliative and supportive care, aiming to help minimise the cost of care and improve the quality of care [2]. For instance, the models of standalone clinics, embedded clinics, telehealth-based palliative care, and enhanced primary-care-based palliative services have respectively been supported by positive evaluation results [2–5]. Regardless of the model used, a competent interdisciplinary team, as well as efficient and early referral, are paramount for delivering successful patient-centred services [2].

### International trend of integrating traditional Chinese medicine in oncology care

The recent concept of integrative oncology (IO) lays the foundation for increasing the type of healthcare professionals involved in oncological care delivery. IO is a patient-centred, evidence-informed approach that advocates the combined use of traditional and complementary medicine and conventional medicine to optimise the health, quality of life and clinical outcomes of patients with cancer, in addition to empowering them to become active participants in their care before, during, and beyond cancer treatment [6]. Since 2003, the Society for Integrative Oncology has synthesised evidence supporting the effectiveness and safety of selected modalities and published relevant clinical practice guidelines with the National Cancer Institute and the American Society of Clinical Oncology [7]. Traditional Chinese medicine (TCM), comprising Chinese herbal medicine, acupuncture, and Chinese massage, is one of the major modalities in IO, with acupuncture being recommended in guidelines for pain management and supportive care for breast and lung cancers [8–10].

### Interprofessional collaboration in implementing integrative oncology

Successful implementation of IO relies on seamless interprofessional collaborations between healthcare professionals, including traditional and complementary medicine practitioners, biomedically trained doctors (BMDs), nurses, and health service administrators [11]. Recently, a mixed-methods systematic review synthesised international experiences regarding the implementation determinants of IO service in conventional cancer care settings [12]. From the included studies, this systematic review summarised that the common barriers to IO interprofessional collaborations included: the lack of IO knowledge regarding safety, funding, and healthcare professionals’ receptiveness towards IO. Common implementation facilitators were the dissemination of evidence on the clinical benefits of using IO, the equipping of professionals with relevant IO skills, and the provision of a supportive organisational climate [12]. While this systematic review provides a snapshot of implementation determinants on IO services worldwide, lessons learnt might not be entirely applicable to specific healthcare systems (e.g., mixed public–private health system in Hong Kong with parallel practices of TCM and Western medicine) due to different local contexts in areas of services delivery, professional regulation, patient preferences, and financial arrangements.

### Regulation, education, and provision of traditional Chinese medicine services in Hong Kong

In Hong Kong, TCM has been a key player in providing routine outpatient care and filling the effectiveness gaps of Western (or conventional) medicine for more than a century [13]. TCM institutionalisation took place in 1999 via the enactment of the Chinese Medicine Ordinance, and the Chinese Medicine Council of Hong Kong (CMCHK) was formed under the Ordinance as a statutory body for licensing and regulating TCM practitioners (TCMPs) and any TCM-related activities [13]. From 2003 to 2014, a total of 18 Chinese Medicine Centres for Training and Research (CMCTRs) were established one after another by the Hospital Authority (the statutory body providing most tax-funded healthcare services in Hong Kong) [14], aiming to provide limited volume of TCM outpatient services and offer training placements for local TCM graduates. Each of these CMCTRs is operated through a tripartite collaboration between the Hospital Authority, a non-governmental organisation, and a local university [14]. The private sector, comprised of TCMPs from private practices, non-governmental organisations, and registered charities, remains the main provider of services. For education, the Hong Kong Baptist University, Chinese University of Hong Kong, and University of Hong Kong are responsible for providing undergraduate and postgraduate education and continuous medical education (CME) activities via their School of Chinese Medicine [15]. Various TCM professional societies (termed ‘accredited administrators’ by the CMCHK) also play an essential role in hosting CME activities and documenting TCMPs’ participation in all these activities to support their three-year licence renewal cycle [16].

### Policies for promoting interprofessional collaborations between traditional Chinese medicine and conventional medicine clinicians

At the policy level, the Health Bureau of the Hong Kong Government is responsible for developing, coordinating, and promoting all healthcare and health-related services [17]. It commissions the Chinese Medicine Unit to plan and oversee TCM development in the territory [18]. Despite its popularity in Hong Kong, TCM was not introduced into inpatient settings until 2014 when the Hospital Authority initiated the ‘Integrated Chinese–Western Medicine’ (ICWM) pilot programme, providing stroke rehabilitation, musculoskeletal pain management, and cancer care services (i.e., IO services in the Hong Kong context were that TCM is used alongside conventional medicine) in public hospitals [19]. The programme offers affordable IO inpatient healthcare services and encourages collaborations between TCMPs and BMDs through co-formulating patient management plans [20]. Nonetheless, since these IO services are confined to inpatient care, none of the abovementioned outpatient oncological service delivery models is adopted in Hong Kong to meet the demand in the community as per international trends.

### Policy context of promoting outpatient integrative Chinese–Western medicine services in oncology

Since 2017, the Hong Kong Health Bureau has been in charge of forming the Steering Committee on Primary Healthcare Development to comprehensively review the planning of primary healthcare services (including TCM services) and draw up the Hong Kong Primary Healthcare Blueprint [21]. One of the Committee’s major contributions is the establishment of District Health Centres (DHCs) in all districts of Hong Kong [21]. Located in seven districts thus far, DHCs are the hubs for enhancing the coordination among local medical and social service providers. The goal of DHCs is to deliver comprehensive, sustainable, and people-centric services at the district level through public–private partnerships (PPPs) [22]. Their scope of practice also includes chronic disease management and community rehabilitation [23]. Partially driven by the projected increase in annual cancer incidence, the Health Bureau is proposing to extend IO services for patients yet to reach to palliative care stage [24, 25]. Meanwhile, according to the Hong Kong Primary Healthcare Blueprint, there will be a strengthening of DHCs’ role to coordinate the referrals between private TCMPs, private BMDs, and other allied healthcare professionals [24], aiming to fill the service gap in outpatient settings.

However, years of experience from the Hospital Authority-based inpatient IO programme might not be transferable to outpatient IO services where interprofessional collaborations would likely rely on the loose networks between DHCs, private TCMPs, and private BMDs. Under this context, the following three Implementation Questions need to be answered with Hong Kong stakeholders’ insights:

- Implementation Question 1: What are the local implementation determinants of outpatient IO service delivery and utilisation? (Answered in our previous work [26])
- Implementation Question 2: What is the most preferred model for DHC-based outpatient IO service delivery and utilisation? (Will be answered in this study)
- Implementation Question 3: Under the preferred model, what are the best implementation interventions (IIs) to address the identified implementation determinants? (Will be answered in this study)

Building upon our previous work [26], this study aims to incorporate the perspectives of local providers and users into the ongoing development of an outpatient IO service delivery model tailored to the context of Hong Kong, laying the foundation for future policy development. This series of research, comprising both exploratory and contextual interviews as well as a Delphi study, is also expected to provide a robust framework for similar healthcare service delivery model investigations worldwide.

## Methods

**Fig. 1:**
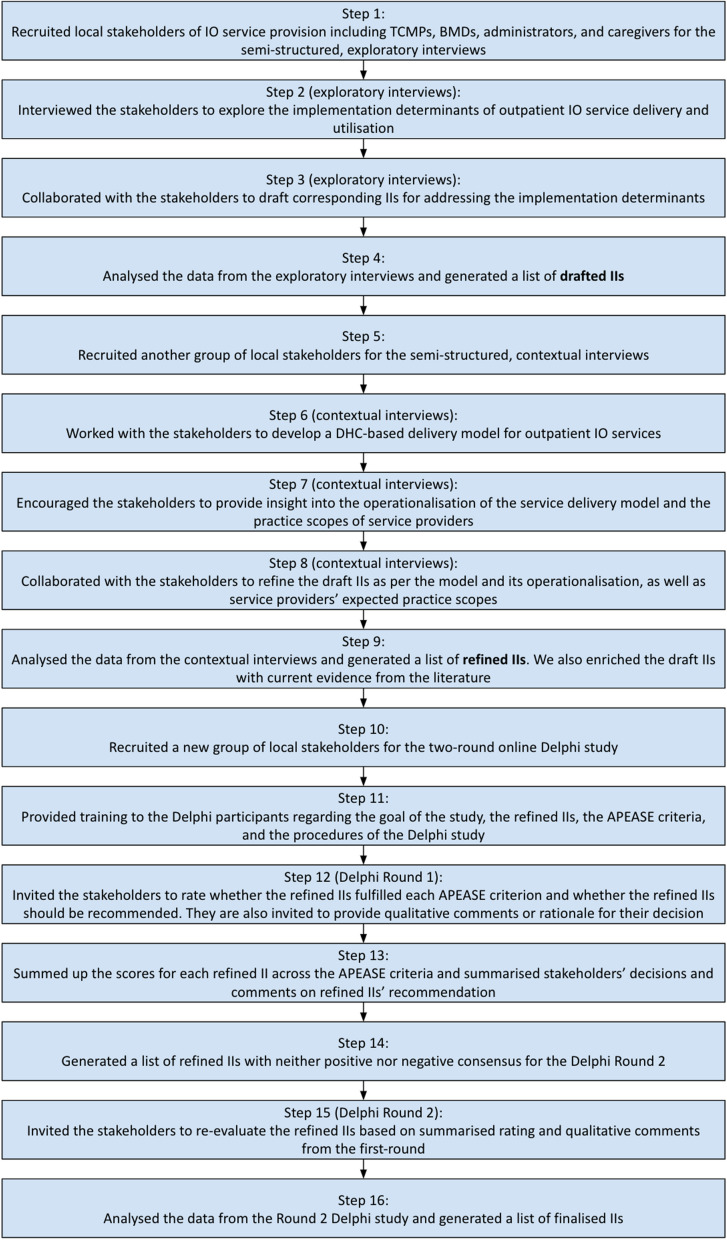
Workflow of generating a list of local stakeholder-recommended implementation interventions for promoting District Health Centre-based outpatient integrative oncology service delivery. APEASE: Acceptability, practicability, effectiveness, affordability, side effects or spill-over effects, and equity; BMD: Biomedically trained doctor; DHC: District Health Centre; IO: Integrative oncology; TCMP: Traditional Chinese medicine practitioner

Our work consists of 16 steps as follows [Fig. 1]:

To answer Implementation Question 1, we conducted the semi-structured, exploratory interviews with 31 potential stakeholders of IO in Hong Kong from February to May 2022. These interviews aimed to identify the possible barriers and facilitators to outpatient IO service delivery and utilisation. Based on these findings, we drafted corresponding IIs (referred to as ‘implementation strategies’ in that publication) to address the identified determinants. Findings of the exploratory interviews are reported elsewhere [26].

In the current manuscript, we report subsequent research work which aims to answer Implementation Questions 2 and 3, with a particular focus on the Hong Kong DHC-based IO service context:

For Implementation Question 2: To perform semi-structured, contextual interviews (from November 2022 to January 2023), which aims to develop a preferred model that would enable DHC-based IO interprofessional collaborations at the district level, and to refine the draft IIs produced in the exploratory interviews [26] with reference to the preferred model and current evidence (Steps 5 to 9);

For Implementation Question 3: To conduct a Delphi survey from April to May 2023 to contextualise and finalise the IIs via stakeholder consensus, with explicit reference to the DHC-based IO service delivery model (Steps 10 to 16).

Given the novelty of the proposed DHC-based outpatient IO service provision, it is essential to consider the local healthcare landscape when formulating the preferred service model and corresponding IIs. Therefore, we designed Steps 5 to 16 of this study based on the Basel Approach for coNtextual ANAlysis (BANANA) [27], which guides how to adopt theories, models, or frameworks and empirical evidence to conduct a contextual analysis for successful design of IIs.

### Choosing a framework to guide contextual analysis

Instead of a single theoretical framework, our contextual analysis was underpinned by the Hong Kong Primary Healthcare Blueprint, the statutory functions and responsibilities of DHCs, the findings from a systematic review of the implementation determinants of IO services [12], and the list of 36 draft IIs emerged from our exploratory interviews [26]. These provided a comprehensive framework consisting of information on Hong Kong’s healthcare policy directions, international experiences, and local stakeholders’ opinions to feed into further data collection and analysis in Steps 5 to 9.

### Using empirical evidence derived from local stakeholders to identify implementation context and contextual factors

In Steps 5 to 9, we conducted semi-structured, contextual interviews with 11 local stakeholders and users to develop a DHC-based delivery model for outpatient IO services and substantiate the draft IIs [26] with current literature. These findings were then fed into the Delphi study mentioned in the subsequent section. Each interview consisted of two stages:

Stage 1 (Steps 5 to 7): We worked collaboratively with the stakeholders to define the practice scope of each service provider (i.e., DHC nurses and other staff members, private TCMPs, private BMDs, and pharmacists) based on the assumption that DHCs would be the pivot for outpatient IO service delivery in the community. Then, we invited the stakeholders to provide insight into the operationalisation of this service delivery model in terms of the expected functions of DHCs, the pathway for DHC-led interprofessional collaborations and referrals between private TCMPs and private BMDs, and the concerns about this service delivery model.

Stage 2 (Steps 8 to 9): We asked the stakeholders to refine and enrich the list of 36 draft IIs [26] based on the discussed service delivery model, its operationalisation, and the expected practice scopes of service providers. Subsequently, we required them to identify the (1) actions, (2) targets of the actions, (3) temporality, and (4) affected implementation outcomes of each refined II based on the *Proctor *et al. approach [28]. Box S1 described details of the 36 IIs designed to support the implementation of the model.

### Assessing context and determining contextual factors’ relevance to implementation interventions

#### Framework adopted in the Delphi study

Based on the findings from the contextual interviews, we conducted an online Delphi survey with local stakeholders of outpatient IO services. This two-round study aimed to encourage local stakeholders to consider the APEASE criteria which determine whether particular refined IIs should be recommended in the local context [29]. This process allowed the participants to consider whether the refined IIs would fit the context of DHC for facilitating the delivery of outpatient IO service in Hong Kong. The six APEASE criteria include [30]:

1. Acceptability – “How far is the II acceptable to the local stakeholders?”
2. Practicability – “Can the II be rolled out at scale within the context of the DHC-based service delivery model?”
3. Effectiveness or cost-effectiveness – “How effective or cost-effective is the II in facilitating DHC-based outpatient IO services in Hong Kong?”
4. Affordability – “How far can the II be afforded when delivered at the scale intended?”
5. Side effects or spill-over effects – “What are the chances that the II will lead to unintended outcomes?”
6. Equity – “How far will the II increase or decrease differences between advantaged and disadvantaged sectors of society?”

The APEASE criteria provide policymakers and other stakeholders with a comprehensive and structured framework to evaluate interventions, facilitating strategic judgments regarding their appropriateness within specific contexts based on six key dimensions [29]. Furthermore, the APEASE framework fosters a holistic approach to decision-making by integrating scientific evidence with practical considerations [29]. This approach not only supports the design and implementation of contextually relevant interventions but also bridges the gap between evidence-based research and real-world practice, thereby enhancing the likelihood of achieving sustainable and impactful outcomes [29]. In the Delphi study, the APEASE criteria served as a theoretical framework, providing participants with a theoretical basis for judging whether they would recommend each of the proposed IIs.

#### Purposive sampling of Delphi participants

We purposefully sampled two groups of local stakeholders in this study (Step 10). The first group included ten healthcare professionals (i.e., TCMPs, BMDs, nurses, administrators, and pharmacists) and caregivers. Those healthcare professionals must have experience delivering IO services in Hong Kong, while the caregivers must have experience caring for cancer patients who had utilised local IO services. The second group would involve 11 healthcare professionals and caregivers without relevant experience. Such purposive sampling of a total of 21 participants with or without IO experience helped us identify implementation challenges during the scaling up or maintenance of existing services, or during the establishment of new services, respectively.

#### Preparation of online training materials

We produced a narrated presentation to provide the Delphi participants with an overview of the refined 36 IIs (product of Stage 2) and their actions, targets of the actions, temporality, affected implementation outcomes, and justifications [28]. We also offered a detailed explanation of the DHC-based service delivery model (product of Stage 1), the practice scopes of each service provider, and the concerns raised by the participants in the exploratory interviews. In addition, the training package included information regarding this project, the APEASE criteria, and the procedures of the online Delphi study (Step 11). We encouraged the stakeholders to read all the materials and raise any questions before they attempted the Delphi study questionnaire.

#### Delphi study data collection and analysis

We sent the first-round Delphi questionnaire to all stakeholders via email (Step 12). In the survey, they were invited to provide their judgement on whether the refined IIs fulfilled each APEASE criterion by giving 0 (the II not fulfilling the criterion) or 1 score (the II fulfilling the criterion). In other words, the highest score for each refined II would be 126 (21 stakeholders * 6 criteria). Then, by referring to their own APEASE rating, each participant was asked to consider whether the refined IIs should be recommended, using a four-point Likert scale (‘recommend against this intervention’, ‘suggest not this intervention’, ‘suggesting this intervention’, or ‘recommending this intervention’). We also elicited qualitative comments regarding the decisions made by all participants.

For data analysis of Delphi Round 1 (Step 13), we summed up the scores for each refined II across the six APEASE criteria and reported the stakeholders’ recommendation rating for each of the refined IIs using medians and interquartile ranges (IQRs). Following the cut-off agreement in Delphi studies [31], the consensus threshold was set at 70% for this study. In other words, if ≥ 70% of the participants recommended or suggested a refined II, that particular II was regarded as reaching a positive consensus from the panel. Otherwise, a negative consensus on the II would be reached if ≥ 70% of stakeholders suggested against or recommended against it. We also analysed the qualitative comments on the refined IIs and presented those that were representative.

If a refined II reached neither positive nor negative consensus in Delphi Round 1, we would re-evaluate them in a Round 2 Delphi survey with the same group of participants (Steps 14 to 16). Together with the Delphi survey, we would present anonymous assessment results for each refined II, its APEASE scores, and relevant qualitative comments to the stakeholders alongside their own ratings from the first round. In Delphi Round 2, data analysis was conducted similar to Delphi Round 1.

Before the contextual interviews and the conduct of the Delphi study, we obtained ethical approval from the Survey and Behavioural Research Ethics Committee of the Chinese University of Hong Kong (Reference number: SBRE-21–0164). Written informed consent from all interviewees and Delphi participants was sought prior to data collection.

## Results

### What is the most preferred model for DHC-based outpatient integrative oncology service delivery and utilisation? (Implementation Question 2)

**Fig. 2:**
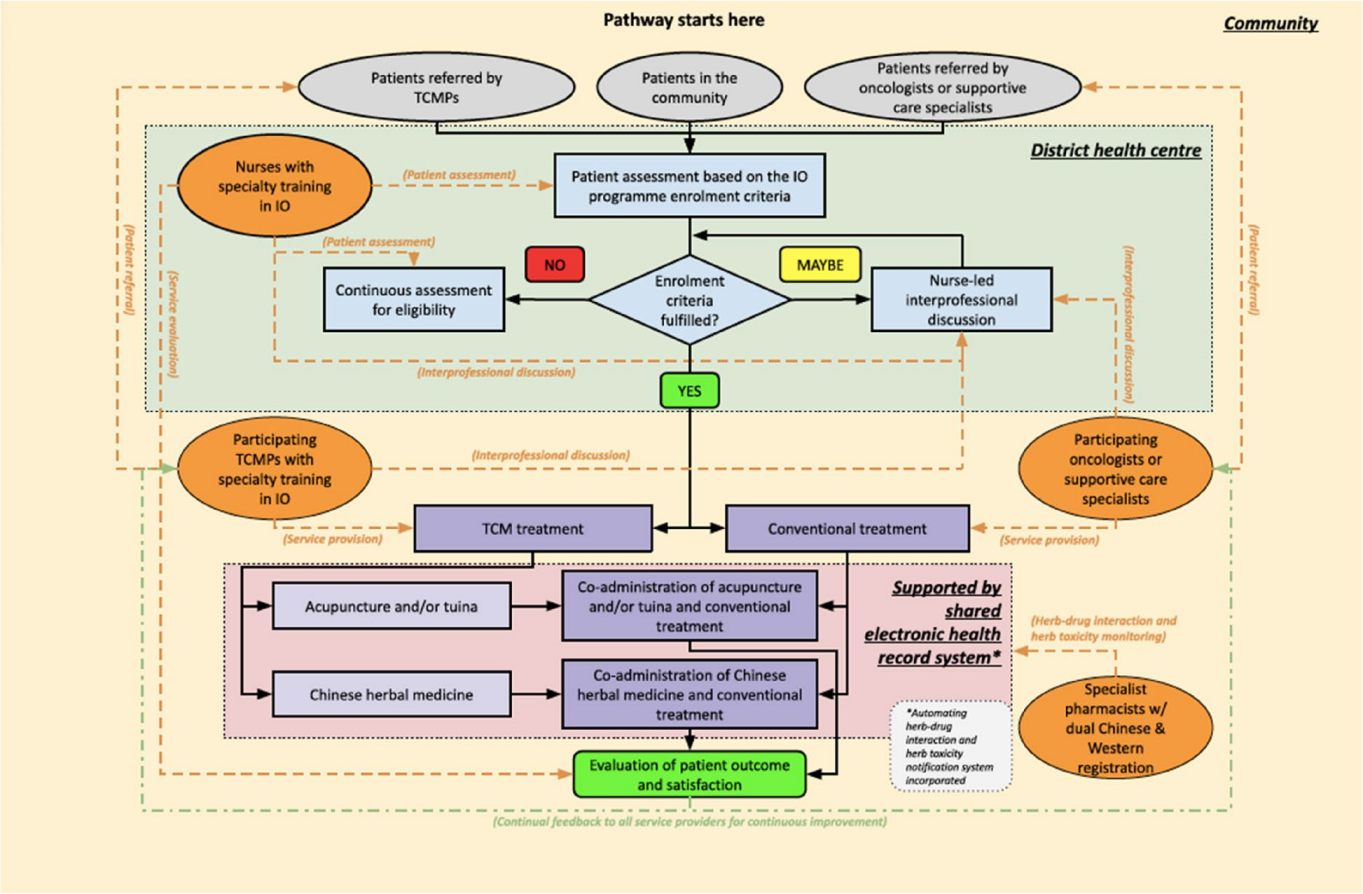
Proposed interprofessional collaboration framework for delivering integrative oncology services in the community. IO: Integrative oncology; TCM: Traditional Chinese medicine; TCMP: Traditional Chinese medicine practitioner

Table 1 presents the demographic characteristics of the stakeholders participated in the contextual interviews. Figure 2 shows the DHC-based outpatient IO service delivery model developed collaboratively with the stakeholders. In this proposed model, the DHCs would play a central role under the leadership of an IO specialist nurse. They would be expected to initiate, arrange, coordinate, and evaluate the delivery of outpatient IO services and facilitate the communications between private TCMPs and private BMDs participating in the programme.

**Table 1:** Characteristics of 11 local stakeholders participated in the contextual interviews

Characteristics	Number of participant (%)
Female	9 (81.8)
Age group (years)	
< 30	0 (0.0)
31 to 45	5 (45.5)
46 to 60	4 (36.4)
> 61	2 (18.2)
Role	
Traditional Chinese medicine practitioner	0 (0.0)*
Biomedically trained doctor	1 (9.1)
Nurse	2 (18.2)
Healthcare administrator	4 (36.4)
Caregiver	4 (36.4)
Years of practice or caregiving experience	
< 5	2 (18.2)
6 to 10	1 (9.1)
11 to 15	2 (18.2)
16 to 20	3 (27.3)
> 21	3 (27.3)

^*^The two interviewers leading the discussions are registered traditional Chinese medicine practitioners in Hong Kong

Regardless of their referral status, all patients who seek to enrol in the programme would receive an assessment conducted by IO specialist nurses against relevant enrolment criteria at the DHCs. If qualified, he/she would be assigned and referred to a private TCMP with specialty training in IO and a private BMD (oncologist, supportive care specialist, or family physician with relevant training). For those failing to fulfil the enrolment criteria, he/she would be placed on a waiting list and asked to attend subsequent assessments for eligibility. For borderline cases, enrolment would be re-assessed during a nurse-led interprofessional case discussion between the three healthcare professionals on his/her eligibility.

Once the referrals are completed, the patient would receive TCM treatments (i.e., acupuncture, tuina (Chinese massage), and Chinese herbal medicine) by the private TCMP and conventional medical treatments by the private BMD in the community, in a coordinated manner. The conventional medical treatments provided would be determined by the BMDs. The TCMP and BMD in charge would be able to collaborate on the shared electronic health record system. Specifically, the system would allow them to have an overview of all diagnostic test and physical examination results, current TCM and conventional treatments prescribed, and the patient’s complete medical history. It would also enable them to communicate via the direct message function securely.

To enhance patient safety, an automated surveillance system would be incorporated into the shared electronic health record system to detect potential negative herb–drug interactions and herbal toxicities. Specialist pharmacists with dual registration in TCM and conventional pharmacy would be responsible for monitoring herb–drug interactions and herbal toxicities on the notification system, updating the system database with the latest clinical evidence, and providing consultation services to all TCMPs and BMDs participating in the IO programme. Apart from case coordination, the IO specialist nurses at the DHCs would evaluate patient outcomes and satisfaction with the IO services and provide continual, individual feedback to the TCMPs and BMDs for continuous improvement.

Tables S1 to S5 described details of the 36 IIs designed to support the implementation of the model.

### Under the preferred model, what are the best implementation interventions to address the identified implementation determinants? (Implementation Question 3)

We invited 22 local stakeholders who did not participate in the exploratory or contextual interviews to constitute the Delphi panel. One of them failed to complete the first Delphi survey, and therefore, the response rate for this study was 95.5%. Table 2 presents the demographic characteristics of the 21 participants.

**Table 2:** Characteristics of local stakeholders participated in the Delphi study

Characteristics	Number of participant (%)
Female	9 (42.9)
Age group (years)	
< 30	1 (4.8)
31 to 45	7 (33.3)
46 to 60	8 (38.1)
> 61	5 (23.8)
Role	
Traditional Chinese medicine practitioner	4 (19.0)
Biomedically trained doctor	4 (19.0)
Nurse	4 (19.0)
Healthcare administrator	4 (19.0)
Caregiver	3 (14.3)
Pharmacist	2 (9.5)
Years of integrative oncology experience as service provider or caregiver (*n* = 10)	
≤ 5	7 (70.0)
6 to 10	1 (10.0)
> 10	2 (20.0)

Delphi Round 1 achieved positive consensus on 35 out of 36 IIs. These IIs had a median recommendation rating of 3 (out of 4), with IQRs of 0 to 1. The low IQRs indicate little data dispersion and, thus, indicate low variability among stakeholders on the recommendation. The percentages of stakeholders who agreed to suggest or recommend these 35 IIs ranged from 76.2% to 100%, eight of which achieved consensus from all stakeholders [Table 3]. As most of the IIs were recommended, we decided not to continue with the Delphi Round 2.

**Table 3:** Delphi participants’ agreement and APEASE judgement on each implementation intervention

Implementation intervention	Agreement		Consensus	Individual APEASE score* (out of a total of 21)						Sum of individual APEASE score
	Median (IQR) on five-point Likert scale	%								
				Ac	P	Ef	Af	S	Eq	
*Recommended by the local stakeholders (*≥ *70% agreement)*										
1. Publicising the regulatory mechanisms of TCM practice	4 (1)	85.7	Yes	21	20	18	18	16	19	112
2. Launching a specialised healthcare voucher scheme for IO services	3 (1)	76.2	Yes	16	18	13	18	13	18	96
3. Expanding the coverage of IO services in the Hong Kong Voluntary Health Insurance Scheme	4 (1)	95.2	Yes	19	20	18	19	16	19	111
4. Setting performance indicators for the number of appropriate IO referrals	3 (1)	85.7	Yes	19	17	17	19	18	18	108
5. Setting performance indicators for IO clinical pathway and service process compliance	3 (1)	81.0	Yes	21	16	17	16	18	19	107
6. Establishing assessment criteria to appraise the clinical effectiveness of, and patient satisfaction towards, IO services	3 (1)	95.2	Yes	21	19	19	17	19	19	114
7. Allocating additional resources to improve the research capacity on TCM and IO safety and effectiveness	4 (1)	90.5	Yes	21	21	19	20	20	20	121
8. Developing DHCs as the coordinators of district-based outpatient IO services	3 (0)	81.0	Yes	17	17	15	17	16	16	98
9. Supporting the establishment of an IMCGC	3 (1)	90.5	Yes	20	20	18	19	19	18	114
10. Developing formal referral mechanisms between DHCs, TCM clinics, and BMD clinics	4 (1)	100	Yes	21	21	21	21	20	20	124
11. Developing IO clinical pathways and service processes	3 (1)	90.5	Yes	21	18	19	17	19	20	114
12. Organising continuing professional development programmes on IO services and encouraging all healthcare professionals to participate	3 (1)	100	Yes	19	20	21	19	19	17	115
13. Drafting accreditation criteria for IO service providers	3 (1)	95.2	Yes	21	19	19	19	17	19	114
14. Establishing IO specialty training schemes for TCMPs and DHC nurses, and improving remuneration packages upon satisfactory completion of training and satisfactory performance	3 (1)	81.0	Yes	20	17	16	16	16	16	101
15. Planning sustainable clinical training programmes for IO services	3 (1)	100	Yes	21	19	18	20	20	20	118
16. Developing regulations to clarify the duties and legal responsibilities of IO healthcare professionals	3 (1)	100	Yes	20	20	21	20	21	21	123
17. Developing an accessible online information platform for herb–drug interactions and Chinese herbal medicine safety for all healthcare professionals	3 (1)	100	Yes	18	18	18	19	20	20	113
18. Recruiting pharmacists with dual qualifications in TCM and conventional pharmacy for professional support	3 (1)	81.0	Yes	17	15	15	17	20	19	103
19. Integrating clinical pathways and service processes into shared electronic health record systems	3 (1)	85.7	Yes	20	14	17	15	16	18	100
20. Elucidating TCM’s modern development to all healthcare professionals	4 (1)	85.7	Yes	21	16	17	18	20	21	113
21. Providing all healthcare professionals with updated research evidence on IO safety and effectiveness	4 (1)	100	Yes	21	18	21	20	21	21	122
22. Incorporating TCMP–BMD–nurse interprofessional communication skills training into undergraduate education	4 (1)	90.5	Yes	20	18	19	20	20	18	115
23. Recruiting specialist nurses to coordinate care in IO services	3 (0)	76.2	Yes	17	20	16	16	18	19	106
24. Recruiting administrative staff to assist in the operation of IO services	3 (1)	85.7	Yes	18	18	17	18	17	18	106
25. Developing a list of accredited IO service healthcare providers in the district to facilitate IO service delivery	3 (1)	90.5	Yes	21	18	18	16	18	18	109
26. Organising team-building activities for IO healthcare professionals in the district	3 (1)	76.2	Yes	16	17	13	16	16	15	93
27. Providing reports on clinical outcomes and patient satisfaction to healthcare providers	3 (1)	100	Yes	20	20	20	21	20	19	120
28. Developing a shared electronic health record system	3 (1)	90.5	Yes	17	18	17	18	17	19	106
29. Delivering collaborative clinical training programmes for IO services	3 (1)	85.7	Yes	19	18	18	16	19	18	108
30. Inviting Chinese Mainland TCM experts to set up training sites	3 (0)	85.7	Yes	17	17	15	14	18	20	101
31. Inviting patients and caregivers to share their experience using IO services	3 (1)	95.2	Yes	19	20	17	18	19	20	113
32. Explaining the fees of IO services and relevant subsidy schemes available to patients and caregivers	3 (1)	100	Yes	21	21	20	21	20	21	124
33. Elucidating TCM’s modern development to the general public	3 (1)	81.0	Yes	20	19	15	19	19	19	111
34. Promoting IO services to the general public	3 (1)	90.5	Yes	20	17	18	20	20	19	114
35. Organising campaigns to promote IO services as a key healthcare initiative endorsed by the government and the public	3 (1)	85.7	Yes	20	19	17	17	18	19	110
*Not recommended by the local stakeholders (*< *70% agreement)*										
• Establishing a subsidy scheme for confirmed cancer patients who are not eligible for the coverage of the Voluntary Health Insurance Scheme nor the proposed specialised healthcare voucher scheme	3 (1)	66.7	No	13	14	10	16	14	16	83

APEASE: *Ac* Acceptability, *P* practicability, *Ef* effectiveness, *Af* affordability, *S* side effects or spill-over effects, and *Eq* equity, *BMD* Biomedically trained doctor, *DHC* District Health Centre, *IO* Integrative oncology, *IQR* Interquartile range, *TCM* Traditional Chinese medicine, *TCMP* Traditional Chinese medicine practitioner

^*^Calculation of individual APEASE score: For each domain, a stakeholder responded ‘satisfied’ for the satisfaction of the corresponding APEASE domain for an implementation intervention count as 1 APEASE score. The individual APEASE score is the total sum of the ‘satisfied’ response for an APEASE domain

#### Rating on APEASE criteria for the recommended implementation interventions

Affordability might be the most critical criterion in determining whether to recommend a specific II because 20 out of 21 stakeholders would give a positive response over this criterion should they recommend the II. Practicability, spill-over effects, and equity might also be essential, considering that 19 stakeholders would answer positively for these criteria should they recommend an intervention. Table 3 lists the median stakeholders’ ratings for each APEASE criterion across the recommended IIs.

#### Qualitative comments on the recommended implementation interventions

Table 4 presents the qualitative comments from the Delphi participants. Only a few IIs were commented on, and the main common concerns were the lack of manpower and time to roll out the recommended IIs. Specifically, the potential stakeholders expressed that dedicated staff would be necessary to monitor and investigate overcharging cases should outpatient IO services be covered by voluntary health insurance plans. Pharmacists with dual qualifications in TCM and conventional pharmacy and specialist nurses are scarce and might not fulfil the demand for outpatient IO services. Healthcare professionals might not have time to participate in relevant continuing professional development programmes or team-building activities. Table 5 provides further information regarding the concerns stated by the stakeholders.

**Table 4:** Delphi participants’ comments on the implementation interventions with positive consensus

Recommended implementation interventions^*^	Reasons for recommending
*Implementation intervention 2:* Launching a specialised healthcare voucher scheme for IO services	It is worth providing financial support as patient advocates have come across numerous clients with financial hardship seeking support for their TCM treatments
*Implementation intervention 28:* Developing DHCs as the coordinators of district-based outpatient IO services	This intervention would fully utilise the capacity of DHCs in promoting accessible access to health services This intervention would provide a golden opportunity for the Hong Kong Government to promote outpatient care
*Implementation intervention 14:* Establishing IO specialty training schemes for TCMPs and DHC nurses, and improving remuneration packages upon satisfactory completion of training and satisfactory performance	This would be a promising human resource model for the future development of integrative medical services in Hong Kong
*Implementation intervention 17:* Developing an accessible online information platform for herb–drug interactions and Chinese herbal medicine safety for all healthcare professionals	Despite the challenges ahead, we must take the first step to realise the modernisation of TCM and improve the safety of treatments
*Implementation intervention 20:* Elucidating TCM’s modern development to all healthcare professionals	It is important to promote modern developments TCM to BMDs and nurses, especially regarding the application of existing clinical evidence, to increase their acceptability and willingness to collaborate
*Implementation intervention 21:* Providing all healthcare professionals with updated research evidence on IO safety and effectiveness	This intervention should be strongly supported as one of the essential measures to strengthen the role of integrative medicine in the health system
*Implementation intervention 26:* Organising team-building activities for IO healthcare professionals in the district	This intervention would improve the communication between healthcare professionals and encourage mutual support
*Implementation intervention 28:* Developing a shared electronic health record system	There are numerous obstacles in upscaling the Hong Kong Electronic Health Record Sharing System. Incorporating TCM systems into it would be difficult, but it must be done sooner or later as integrative medicine becomes more mainstream
*Implementation intervention 30:* Inviting Chinese Mainland TCM experts to set up training sites	Learning from other health systems, including the practices in China Mainland, would provide opportunities to accelerate service model development in Hong Kong

*BMD* Biomedically trained doctor, *DHC* District Health Centre, *IO* Integrative oncology, *TCM* Traditional Chinese medicine, *TCMP* Traditional Chinese medicine practitioner

^*^Only included recommended implementation interventions that were commented by the stakeholders

**Table 5:** Delphi participants’ concerns regarding the implementation interventions with positive consensus

Recommended implementation interventions^*^	Concerns
*Implementation intervention 1:* Publicising the regulatory mechanisms of TCM practice	The purposes of such regulatory mechanisms should be transparent, or else they would attract irrelevant or false allegations
*Implementation intervention 3:* Expanding the coverage of IO services in the Hong Kong Voluntary Health Insurance Scheme	Manpower is necessary for monitoring and investigating overcharging cases due to the expansion of insurance coverage
*Implementation intervention 4:* Setting performance indicators for the number of appropriate IO referrals	If the performance targets were set to high, the healthcare professionals might not be able to achieve it, and many would choose not to continue their participation
*Implementation intervention 5:* Setting performance indicators for IO clinical pathway and service process compliance	
*Implementation intervention 12:* Organising continuing professional development programmes on IO services and encouraging all healthcare professionals to participate	Healthcare professionals might not have time to participate in such activities
*Implementation intervention 14:* Establishing IO specialty training schemes for TCMPs and DHC nurses, and improving remuneration packages upon satisfactory completion of training and satisfactory performance	If the training content has demanding standard, TCMPs and DHC nurses might not participate in such specialty training despite an increase in salary after completion
*Implementation intervention 18:* Recruiting pharmacists with dual qualifications in TCM and conventional pharmacy for professional support	Pharmacists in Hong Kong holding both qualifications are scarce
*Implementation intervention 19:* Integrating clinical pathways and service processes into shared electronic health record systems	Resources should be allocated to help the older TCMPs who have relatively low computer literacy
*Implementation intervention 23:* Recruiting specialist nurses to coordinate care in IO services	Drawing too many specialist nurses from the public hospitals to the community might worsen the current nurse shortage problem
*Implementation intervention 26:* Organising team-building activities for IO healthcare professionals in the district	Surveys should be conducted to probe whether healthcare professionals are interested in such activities and whether they have time to participate

*BMD* Biomedically trained doctor, *DHC* District Health Centre, *IO* Integrative oncology, *TCM* Traditional Chinese medicine, *TCMP* Traditional Chinese medicine practitioner

^*^Only included recommended implementation interventions that were commented by the stakeholders

## Discussion

### Summary of findings

Guided by the BANANA framework, we created a list of 35 local stakeholders-recommended, context-relevant IIs for promoting DHC-based outpatient IO service delivery established from exploratory and contextual interviews, as well as the Delphi study. For these recommended IIs, shared concerns about the implementation from stakeholders’ perspectives were mainly related to the lack of manpower in the Hong Kong healthcare system and the lack of time among healthcare professionals to participate in professional development programmes and team-building activities.

### Implications for policy and practice

Interprofessional collaborations between TCMPs and BMDs in cancer care mostly take place in the public sector through the inpatient-focused ICWM programme. Although the government is planning to reinforce DHCs’ role in coordinating private TCM services at the district level, implementing this via a PPP mechanism might encounter policy challenges, given the insubstantial primary care infrastructure in Hong Kong [32, 33]. While the Hospital Authority has ample experience in PPPs, their service scopes are only confined to conventional medical service, and DHCs were not involved [34]. Recently, the first DHC-based PPP programme ‘Chronic Disease Co-Care Pilot Scheme’ was rolled out in 2023, which may serve as a future reference for our proposed IO service delivery model [35, 36]. This scheme is designed as a co-payment mechanism using public funds to purchase conventional primary care services provided by the private sector, aiming to increase the capacity of chronic condition prevention, detection, and management. Under this voluntary scheme, citizens aged ≥ 45 years without a known history of hypertension or diabetes mellitus and who pass the eligibility screening at the DHCs will be assigned to accredited private family physicians for further consultation, medical assessment, and laboratory investigation [35]. Individuals with a diagnosis or high risk of hypertension or diabetes mellitus will then receive a maximum of six consultations per year with appropriate treatments from the assigned family physicians. When necessary, they will be referred to public nurse clinics and private allied healthcare professionals for other procedures. Those without the conditions can also attend health management group activities held by the DHCs. All diagnostic and treatment services offered in this scheme are partially subsidised by the Hong Kong Government. As of February 2024, a total of 503 private family physicians have signed up for this scheme, covering all 18 districts in Hong Kong [35].

However, if similar arrangements are to be adopted in the delivery of outpatient IO services, apart from previous local experiences, policymakers should pay attention to how the arrangement should be tailored to engage the private TCM sector, especially on the seven factors crucial to the sustainability of PPPs [37]: (1) trust between the government and private TCM sectors; (2) clear partnership objectives and roles for all parties involved in Fig. 2; (3) time commitment; (4) candid information regarding risks and benefits of interventions for cancer patients and caregivers; (5) flexibility of contracts with the private sector; (6) incentive of arrangements; and (7) awareness and acceptability of structural changes in power and authority.

From an international perspective, our proposed DHC-based outpatient IO service delivery model resembles the interdisciplinary standalone cancer palliative care clinics model widely adopted in North America [2–5]. In this model, services are delivered by specialist care teams consisting of physicians, nurses, and psychosocial professionals in freestanding clinics within the community instead of in interdisciplinary service departments embedded in hospitals [2–5]. Patients (and their family members), who often have diverse and complex needs, are able to receive comprehensive support in an organised, non-fragmented fashion after triage and internal referral. Yet, unlike the interdisciplinary standalone clinics model, our proposed model is designed around the DHCs, which are already providing services in the districts, and healthcare professionals are not required to work together in specific venues but collaborate via a virtual referral network. Beyond patient screening, the DHC-based IO specialist nurses will have a vital role in reviewing treatment plans, providing feedback to care providers and being both the facilitator and participant in interprofessional communications. Such consolidated nurse-led collaborations ensure that the participating TCMPs and BMDs will have similar expectations and priorities in delivering IO services, and the services provided will be synchronised, timely, and patient-centred [2].

According to a recent systematic review, there are various implementation determinants of IO services in modern healthcare systems [12]. We found that three of the eight IIs that received complete endorsement from local stakeholders (i.e., with 100% consensus) in the Delphi study are related to three common implementation determinants mentioned in the systematic review. The II of providing all healthcare professionals with updated research evidence on IO safety and effectiveness echoes the demand for evidence on the IO clinical benefits. The II of developing accessible online information platforms for herb–drug interactions and Chinese herbal medicine toxicities resonates with the need for evidence on the safety profiles of herbal medicine. The II of organising continuing professional development programmes anchors the request for upskilling healthcare professionals with IO knowledge. Nonetheless, the common IO implementation barriers regarding the lack of funding to set up IO services are considered to be less relevant. Instead, in Hong Kong, financial barriers to IO service access among patients appear to be an important topic, as reflected in the strong support for II of launching specialised healthcare voucher schemes (76.2%). This reflects that local contexts are key in developing appropriate IIs, and referring to international experience alone is probably insufficient.

Future research should aim to capture the perspectives of a broader group of local stakeholders, including policymakers and patients currently receiving or recovered from cancer. Policymakers are essential for formulating the technical and operational details of the proposed delivery model, while patients may provide invaluable insights based on their first-hand experiences with existing IO services. Involving health informaticians may also be highly beneficial, as they can offer critical expertise on the potential risks of district-level interprofessional collaboration, particularly in relation to patient data security and privacy concerns. When planning future public consultations on the optimisation of IO services, relevant authorities are encouraged to use the findings of this study as a scientific foundation. These findings can serve as an evidence-based reference to guide discussions and inform decision-making processes, ensuring that proposed changes are grounded in robust research and tailored to address the specific needs of stakeholders.

### Strengths and limitations

This study successfully applied the APEASE criteria to facilitate consensus-making on IIs for promoting outpatient IO service delivery and utilisation in Hong Kong with the involvement of local stakeholders. This transparent framework allows the stakeholders to have thorough consideration of all IIs from different aspects, including acceptability, practicability, effectiveness/cost-effectiveness, affordability, side effects/spill-over effects, and equity, before making their judgements on II recommendations. In addition, by providing them with a stakeholder-co-created IO service delivery model, the stakeholders were able to base their decisions on a substantial context.

There are several limitations to this study. First, adopting purposive sampling might have resulted in selection bias as the local stakeholders were invited through investigators’ professional networks [38]. However, the impact would be insignificant given that the IIs are unlikely to be endorsed unless local stakeholders with all disciplinary backgrounds reach a consensus. Also, having a high consensus threshold of 70%, agreements were unlikely to be achieved by selection bias alone. Second, we did not include policymakers in the Delphi study or both rounds of interviews, and therefore, our IIs did not consider administrative issues from a top-down perspective. In the future, these groups of stakeholders may be included in tailoring intervention details to streamline the process of intervention implementation. Third, instead of including patients currently receiving cancer care or those who had recovered from cancer, we only invited caregivers to participate in the two studies. As a result, we might have missed valuable feedback directly reflecting the user perspective, which could have provided a more comprehensive understanding of the interventions’ impact and relevance. Fourth, due to the unavailability of relevant data, we were unable to provide participants in the Delphi study with an estimation of the costs associated with developing and operating the proposed delivery model and various IIs. This limitation may have influenced participants’ ability to fully assess the cost-effectiveness and affordability of the IIs, potentially impacting their judgements. Finally, this study is centred on the context of Hong Kong, which means our findings may not be directly applicable to regions with diverse religious and cultural practices. Nevertheless, our approach to co-designing a service delivery model with local stakeholders may serve as a useful reference.

## Conclusions

With local stakeholders, we co-created a nurse-led service delivery model that aims to forge a partnership between the DHCs and TCMPs and BMDs in the private sector to provide outpatient IO services. Using the APEASE criteria, we also established a list of 35 local stakeholder-recommended IIs for promoting outpatient IO service delivery and utilisation under the proposed model. Before implementing the model using the IIs, further research should be conducted to seek opinions from policymakers in terms of the potential administrative determinants around the implementation.

## Supplementary Information

Supplementary Material 1.

## Data Availability

Data is provided within the manuscript or supplementary information files.
