# Supplementary material for: Contextualising implementation interventions for promoting outpatient integrative Chinese–western oncology service delivery and utilisation in Hong Kong: a Delphi study
Source: BMC Complement Med Ther. 2025 Mar 28;25:118. doi: 10.1186/s12906-025-04779-0 (PMC11951825; doi:10.1186/s12906-025-04779-0)
Supplement: Supplementary file 1 — Supplementary Material 1. [file 12906_2025_4779_MOESM1_ESM.docx]

**Box S1.** Interview guide for the semi-structured, contextual interviews

| **Topic** | **Question** |
| --- | --- |
| DHC-based delivery model | - Could you share your views on a DHC-based delivery model for outpatient IO services? In particular, who should be involved in such a model? |
| Practice scopes of service providers | - Could you share your views on the practice scope of DHC nurses within this model? - Could you share your views on the practice scope of other relevant staff members (e.g., administrative officers) within this model? - Could you share your views on the practice scope of private TCMPs within this model? - Could you share your views on the practice scope of private BMDs within this model? - Could you share your views on the practice scope of pharmacists within this model? |
| Operationalisation of the DHC-based delivery model | - Could you share your expectations regarding the functions of DHCs in this model? - Could you share your views on the pathway for DHC-led interprofessional collaborations and referrals between private TCMPs and private BMDs? - What are the concerns of this model? |
| Refinement and enrichment of the 36 draft IIs | - Could you share your views on each of these IIs drafted from our previous interviews? In particular, do you think they would facilitate the DHC-based delivery model we discussed above? - How would you refine these IIs based on the specified practice scopes of service providers? - How would you refine these IIs based on the specified collaboration and referral pathways? |
| *Proctor et al* approach | - Based on the *Proctor et al* approach we previously sent, could you identify the (1) actions, (2) targets of the actions, (3) temporality, and (4) affected implementation outcomes of each refined II? |
| Other | - Do you have any additional ideas you would like to share with us about the DHC-based delivery model, or anything further you would like to add? |

BMD: Biomedically trained doctor; DHC: District Health Centre; II: Implementation intervention; IO: Integrative oncology; TCM: Traditional Chinese medicine; TCMP: Traditional Chinese medicine practitioner.

**Table S1.** Suggested implementation interventions to be delivered by the Hong Kong Health Bureau

| **Domain** | **Publicising the regulatory mechanisms of TCM practice** |
| --- | --- |
| Action(s) | Provides information regarding the regulatory mechanisms on TCM practice via different types of media (e.g., television and social media) |
| Target(s) of the action | The general public |
|  | Knowledge about the regulatory mechanisms on TCM practice in Hong Kong, including how and to whom they can file medical negligence and malpractice complaints to regulatory body (i.e., the Chinese Medicine Council of Hong Kong) |
| Temporality | Continuous |
| Implementation outcome(s) affected | Acceptability; adoption |
| Justification | Evidence suggested that patients believed that lack of regulatory standards limit the scope and penetration of IO services, as the current mechanisms may not guarantee safety and quality.[1] |
| **Domain** | **Launching a specialised healthcare voucher scheme for IO services** |
| Action(s) | Develops and launches IO-specific healthcare vouchers to support cancer patients in need |
| Target(s) of the action | Cancer patients with financial difficulties and the general public |
|  | Cancer patients’ accessibility to outpatient IO services |
|  | Misallocation of resources to those who do not need subsided healthcare services |
| Temporality | Continuous |
| Implementation outcome(s) affected | Adoption |
| Justification | Evidence suggested that people receiving the cash transfers regarded such financial support as helpful in the short term, and sometimes in the long term. The cash allowed them to make more independent healthcare decisions.[2] |
| **Domain** | **Expanding the coverage of IO services in the Hong Kong Voluntary Health Insurance Scheme** |
| Action(s) | Includes outpatient IO services in the basic coverage of the Hong Kong Voluntary Health Insurance Scheme |
| Target(s) of the action | Insurance companies offering the Voluntary Health Insurance Scheme |
|  | Cancer patients’ accessibility to outpatient IO services |
| Temporality | Continuous |
| Implementation outcome(s) affected | Adoption |
| Justification | Evidence suggested that rebates through health insurances or work-related healthcare compensation may increase the concurrent use of integrative treatments.[3] |
| **Domain** | **Formulating subsidy schemes for IO services for patients not entitled to the Voluntary Health Insurance Scheme and the current healthcare voucher scheme** |
| Action(s) | Explores alternative subsidy schemes to ensure all cancer patients can access outpatient IO services |
| Target(s) of the action | Cancer patients’ accessibility to outpatient IO services |
| Temporality | Continuous |
| Implementation outcome(s) affected | Acceptability; adoption |
| Justification | Evidence suggested that rebates through health insurances or work-related healthcare compensation may increase the concurrent use of integrative treatments.[3] |
| **Domain** | **Setting performance indicators for the number of appropriate IO referrals** |
| Action(s) | Establishes performance indicators to monitor and evaluate the number of appropriate referrals between DHC, BMD clinics, and TCM clinics |
| Target(s) of the action | DHC nurses, TCMPs, and BMDs involved in IO service delivery |
|  | DHCs’ and healthcare professionals’ involvement in interprofessional referral and collaboration |
| Temporality | Continuous |
| Implementation outcome(s) affected | Fidelity |
| Justification | Evidence suggested that performance indicators may improve the accountability and quality of care among healthcare professionals.[4] |
| **Domain** | **Setting performance indicators for IO clinical pathway and service process compliance** |
| Action(s) | Establishes performance indicators to evaluate BMDs’ and TCMPs’ compliance with clinical pathways and service processes |
| Target(s) of the action | TCMPs and BMDs involved in IO service delivery |
|  | Healthcare professionals’ clinical professionalism |
| Temporality | Continuous |
| Implementation outcome(s) affected | Fidelity |
| Justification | Evidence suggested that performance indicators may improve the accountability and quality of care among healthcare professionals.[4] |
| **Domain** | **Establishing assessment criteria to appraise the clinical effectiveness of, and patient satisfaction towards, IO services** |
| Action(s) | Develops the assessment criteria for appraising the clinical effectiveness of IO services, as well as patients’ satisfaction towards the services delivered |
| Target(s) of the action | TCMPs and BMDs involved in IO service delivery |
|  | Healthcare professionals’ clinical professionalism |
| Temporality | Continuous |
| Implementation outcome(s) affected | Fidelity; sustainability |
| Justification | Evidence suggested that patients’ feedback and data on clinical effectiveness may be associated with improvements in processes and outcomes of care.[5] |
| **Domain** | **Allocating additional resources to improve the research capacity on TCM and IO safety and effectiveness** |
| Action(s) | Increases financial resources to upgrade TCM research capacity and support research on IO safety and effectiveness |
| Target(s) of the action | Local universities and other research institutions |
|  | Researchers’ resources for conducting TCM and IO research |
| Temporality | Continuous |
| Implementation outcome(s) affected | Sustainability |
| Justification | Evidence suggested that improving research capacity and encouraging healthcare professional to involve in research may positively influence the application of clinical evidence and, therefore, improve clinical outcomes.[6] |
| **Domain** | **Developing DHCs as the coordinators of district-based outpatient IO services** |
| Action(s) | Directs the upscaling of DHCs to be the coordinators of outpatient IO services, facilitating the communications and referrals between TCM clinics and BMD clinics in the district which it is responsible for. |
| Target(s) of the action | DHCs |
| Temporality | Once with constant review |
| Implementation outcome(s) affected | Feasibility; sustainability |
| Justification | Evidence suggested that creating close linkages between existing services (i.e., service coordination) may improve the utilisation and outputs of healthcare delivery.[7] |
| **Domain** | **Supporting the establishment of an IMCGC** |
| Action(s) | Provides resources to develop the IMCGC, the governing body with a multidisciplinary team of experts, responsible for designing IO clinical guidelines and planning the short-, intermediate-, and long-term TCM academic and research developments |
|  | Provides resources to develop the IMCGC as the precursor of the Hong Kong TCM Academy, responsible for designing TCM speciality training programmes |
| Target(s) of the action | Foundation of a sustainable, evidence-informed integrative medicine system in Hong Kong |
| Temporality | Once with constant review |
| Implementation outcome(s) affected | Sustainability |
| Justification | Not available. |

BMD: Biomedically trained doctor; DHC: District Health Centre; IMCGC: Integrative Medicine Clinical Governance Centre; IO: Integrative oncology; TCM: Traditional Chinese medicine; TCMP: Traditional Chinese medicine practitioner.

**Table S2.** Suggested implementation interventions to be delivered by the Integrative Medicine Clinical Governance Centre

| **Domain** | **Developing formal referral mechanisms between DHCs, TCM clinics, and BMD clinics** |
| --- | --- |
| Action(s) | Establishes formal referral mechanisms between DHCs, TCM clinics, and BMD clinics to facilitate efficient interprofessional patient referral |
| Target(s) of the action | The lack of organised interprofessional patient referrals platform |
| Temporality | Once with constant review |
| Implementation outcome(s) affected | Feasibility; fidelity; penetration; sustainability |
| Justification | Evidence suggested that effective and timely patient referrals is crucial to the success of integrative medicine service delivery and utilisation.[8] |
| **Domain** | **Developing IO clinical pathways and service processes** |
| Action(s) | Formulates formal clinical pathways and service processes for healthcare professionals involved in IO service delivery to improve service efficiency, quality, and safety |
| Target(s) of the action | DHC nurses, TCMPs, and BMDs involved in IO service delivery |
|  | The lack of clinical pathways and service processes in outpatient settings |
| Temporality | Once with constant review and regular updating |
| Implementation outcome(s) affected | Fidelity |
| Justification | Evidence suggested that integrative medicine healthcare professionals believed that the availability of clinical guidelines or standard protocols enabled them to routinise service delivery.[9] |
| **Domain** | **Organising continuing professional development programmes on IO services and encouraging all healthcare professionals to participate** |
| Action(s) | Sets up continuing IO professional development programmes for healthcare professionals, regardless of their involvement in IO, and encourage them to participate |
| Target(s) of the action | All healthcare professionals |
|  | Knowledge about IO and the latest developments in the field |
| Temporality | Continuous |
| Implementation outcome(s) affected | Acceptability; adoption; fidelity; penetration |
| Justification | Evidence suggested that continuing training programmes may improve the skills of healthcare professionals in delivering IO services and therefore increase service quality.[10, 11] |
| **Domain** | **Drafting accreditation criteria for IO service providers** |
| Action(s) | Develops central registration system and formulates accreditation criteria (corresponding to professional training and clinical experience) for IO service providers to facilitate patients’ and caregivers’ selection |
| Target(s) of the action | Public recognition of TCMPs and BMDs who are willing to provide IO services |
|  | Quality of IO service providers, including both TCMPs and BMDs |
| Temporality | Once with constant review |
| Implementation outcome(s) affected | Acceptability; feasibility |
| Justification | Evidence suggested that patients prefer healthcare providers with specialist qualification and experience.[12] |
| **Domain** | **Establishing IO specialty training schemes for TCMPs and DHC nurses, and improving remuneration packages upon satisfactory completion of training and** **satisfactory performance** |
| Action(s) | Designs IO specialty training programmes for TCMPs and DHC nurses with local universities and TCM professional societies |
|  | Improving the remuneration packages of the TCMPs and DHC nurses who complete the programmes and achieve satisfactory performance |
| Target(s) of the action | TCMPs’ and nurses’ willingness to provide IO services |
|  | Expertise of TCMPs and nurses in IO service provision |
| Temporality | Continuous |
| Implementation outcome(s) affected | Adoption; fidelity |
| Justification | Evidence suggested that improving the recognition and professional status of healthcare professionals delivering integrative medicine may attract more new blood to the field.[13] |
| **Domain** | **Planning sustainable clinical training programmes for IO services** |
| Action(s) | Explores the development of sustainable clinical training programmes for all TCMPs, BMDs, and nurses with a medium-term aim of improving local training capacity and capability |
| Target(s) of the action | All TCMPs, BMDs, and nurses interested in IO |
|  | Expertise of TCMPs and nurses and collaboration ability of BMDs in IO service provision |
| Temporality | Continuous |
| Implementation outcome(s) affected | Fidelity; sustainability |
| Justification | Evidence suggested that continuing training programmes may improve the skills of healthcare professionals in delivering IO services and therefore increase service quality.[10, 11] |
| **Domain** | **Developing regulations to clarify the duties and legal responsibilities of IO healthcare professionals** |
| Action(s) | Establishes relevant regulations with Hong Kong Chinese Medicine Council, Hong Kong Medical Council, and Hong Kong Nursing Council to reduce healthcare professionals’ concerns and uncertainty during IO service delivery |
| Target(s) of the action | DHC nurses, TCMPs, and BMDs involved in IO service delivery |
|  | IO healthcare providers’ uncertainties on professional liability in IO service delivery |
| Temporality | Once with constant review |
| Implementation outcome(s) affected | Acceptability; adoption |
| Justification | Evidence suggested providing documents to set out the duties and responsibilities of all service providers are always expected by healthcare professionals prior to the delivery of shared care.[14] |
| **Domain** | **Developing an accessible online information platform for herb–drug interactions and Chinese herbal medicine safety for all healthcare professionals** |
| Action(s) | Creates an online information platform for herb–drug interactions and Chinese herbal medicine safety to allow instant information checking for all healthcare professionals, regardless of their involvement in IO, to reduce concerns in clinical practice |
|  | Integrates safety information into existing electronic health record systems to enable automated alerts when contra-indication appears |
| Target(s) of the action | All healthcare professionals |
|  | Healthcare professionals’ concerns about the coadministration of Chinese herbal medicine and conventional drugs |
| Temporality | Once with constant review |
| Implementation outcome(s) affected | Acceptability; adoption |
| Justification | Evidence suggested that the availability of safety evidence is a major contributor to healthcare professionals’ and the public’s acceptance of IO services.[15] |
| **Domain** | **Recruiting pharmacists with dual qualifications in TCM and conventional pharmacy for professional support** |
| Action(s) | Recruits dual-registered pharmacists in TCM and conventional pharmacy to develop and update the online information platforms for herb–drug interactions and Chinese herbal medicine safety |
|  | Recruits dual-registered pharmacists in TCM and conventional pharmacy to provide relevant consultation services on the co-administration of TCM herbs and Conventional drugs |
| Target(s) of the action | All healthcare professionals |
|  | Healthcare professionals’ concerns about the coadministration of Chinese herbal medicine and conventional drugs |
| Temporality | Continuous |
| Implementation outcome(s) affected | Acceptability; adoption; fidelity |
| Justification | Evidence suggested that the availability of safety evidence is a major contributor to healthcare professionals’ and the public’s acceptance of IO services.[15] |
| **Domain** | **Integrating clinical pathways and service processes into shared electronic health record systems** |
| Action(s) | Integrates the formal clinical pathways and service processes into existing electronic health record systems to promote their implementation among IO service providers |
| Target(s) of the action | DHC nurses, TCMPs, and BMDs involved in IO service delivery |
|  | IO healthcare professionals’ compliance with clinical pathways and service processes |
| Temporality | Once with constant review |
| Implementation outcome(s) affected | Feasibility; fidelity |
| Justification | Evidence suggested that on-screen, point of care computer reminders may improve the process adherence of healthcare professionals.[16] |
| **Domain** | **Elucidating TCM’s modern development to all healthcare professionals** |
| Action(s) | Via different types of media (e.g., both traditional and social media), presents and explains TCM development, including the incorporation of conventional medicine teaching in local undergraduate TCM programmes, to all conventional healthcare professionals. This serves to change conventional healthcare professionals’ negative impressions towards TCM and TCMPs, and to boost their confidence in IO |
| Target(s) of the action | All healthcare professionals |
|  | Healthcare professionals’ negative impressions towards TCM and TCMPs |
| Temporality | Continuous |
| Implementation outcome(s) affected | Acceptability; adoption |
| Justification | Evidence suggested that the lack of evidence on effectiveness and safety is a common rhetorical tool against IO services for healthcare professionals. The dissemination of existing evidence should be promoted to act against this problem.[17] |
| **Domain** | **Providing all healthcare professionals with updated research evidence on IO safety and effectiveness** |
| Action(s) | Feeds up-to-date research evidence on IO safety and effectiveness to all healthcare professional, regardless of their involvement in IO, to increase their confidence in, or intention of, providing IO services |
| Target(s) of the action | All healthcare professionals |
|  | Healthcare professionals’ confidence in participating in IO service delivery |
| Temporality | Continuous |
| Implementation outcome(s) affected | Acceptability; adoption |
| Justification | Evidence suggested that the lack of evidence on effectiveness and safety is a common rhetorical tool against IO services for healthcare professionals. The dissemination of existing evidence should be promoted to act against this problem.[17] |
| **Domain** | **Incorporating TCMP–BMD–nurse interprofessional communication skills training into undergraduate education** |
| Action(s) | Works with local universities to design and incorporate specialised undergraduate courses in interprofessional communication to familiarise students with relevant skills |
| Target(s) of the action | Undergraduate medical, TCM, and nursing students |
|  | Students ’ interprofessional communication skills |
| Temporality | Once with constant review |
| Implementation outcome(s) affected | Fidelity; sustainability |
| Justification | Evidence suggested that IO healthcare professionals believed that interdisciplinary communication is essential for the successful implementation of IO services.[1, 18] |

BMD: Biomedically trained doctor; DHC: District Health Centre; IMCGC: Integrative Medicine Clinical Governance Centre; IO: Integrative oncology; TCM: Traditional Chinese medicine; TCMP: Traditional Chinese medicine practitioner.

**Table S3.** Suggested implementation interventions to be delivered by the District Health Centres

| **Domain** | **Recruiting specialist nurses to coordinate care in IO services** |
| --- | --- |
| Action(s) | Employs new or arranges existing specialist nurses to coordinate outpatient IO services in the community, monitor patient referrals, and implement performance evaluation. |
| Target(s) of the action | Shortage in nursing manpower |
| Temporality | Continuous |
| Implementation outcome(s) affected | Fidelity; sustainability |
| Justification | Evidence suggested that IO healthcare professionals believed that lack of capable nurses for delivering IO services limited their capacity to serve new patients and ensure the quality of IO services.[19] |
| **Domain** | **Recruiting administrative staff to assist in the operation of IO services** |
| Action(s) | Employs administrative assistants to handle extra administrative tasks brought on by the implementation of outpatient IO services. |
| Target(s) of the action | Shortage in administrative manpower |
| Temporality | Continuous |
| Implementation outcome(s) affected | Fidelity; sustainability |
| Justification | Evidence suggested that IO healthcare professionals believed that understaffing limited their capacity to provide IO services.[19] |
| **Domain** | **Developing a list of accredited IO service healthcare providers in the district to facilitate IO service delivery** |
| Action(s) | Establishes a list of IO accredited BMDs and TCMPs based on the criteria set by the IMCGC to streamline the selection of providers among patients and caregivers |
| Target(s) of the action | Patients and caregivers |
|  | Knowledge on the availability of IO providers in the community |
| Temporality | Once with constant review |
| Implementation outcome(s) affected | Acceptability; adoption; feasibility, |
| Justification | Evidence suggested that patients prefer healthcare providers with specialist qualification and experience.[12] |
| **Domain** | **Organising team-building activities for IO healthcare professionals in the district** |
| Action(s) | Hosts team-building activities for healthcare professionals involved in IO service delivery in the district to improve their morale and team spirit |
| Target(s) of the action | DHC nurses, TCMPs, and BMDs involved in IO service delivery |
|  | IO healthcare professionals’ teamwork and sense of belonging |
| Temporality | Continuous |
| Implementation outcome(s) affected | Sustainability |
| Justification | Evidence suggested that bridge-building activities may facilitate bonding development among team members involved integrative medicine service delivery.[20] |
| **Domain** | **Providing reports on clinical outcomes and patient satisfaction to healthcare providers** |
| Action(s) | Provides monthly and/or annual reports on clinical effectiveness, safety, and patient satisfaction to IO healthcare professionals involved to enable reflections and quality improvement |
| Target(s) of the action | TCMPs, nurses, and BMDs involved in IO service delivery |
|  | Service quality of IO healthcare professionals |
| Temporality | Continuous |
| Implementation outcome(s) affected | Fidelity |
| Justification | Evidence suggested that patients’ feedback may be associated with improvements in the processes of diagnosis and notation.[5] |
| **Domain** | **Developing a shared electronic health record system** |
| Action(s) | Establishes an electronic health record system accessible by all IO healthcare professionals to facilitate patient information sharing |
| Target(s) of the action | Existing electronic health record systems used by BMDs and TCMPs |
| Temporality | Once with constant review |
| Implementation outcome(s) affected | Adoption ; implementation cost |
| Justification | Evidence suggested that patient chart sharing, preferably on electronic platforms, may foster communications and trust between integrative medicine healthcare professionals and improve efficiency.[21] |
| **Domain** | **Delivering collaborative clinical training programmes for IO services** |
| Action(s) | Sets up professional training sites to deliver sustainable IO clinical training programmes formulated by the IMCGC to ensure the passing on of knowledge |
| Target(s) of the action | All TCMPs, BMDs, and nurses |
|  | Expertise of TCMPs, BMDs, and nurses |
| Temporality | Continuous |
| Implementation outcome(s) affected | Fidelity; sustainability |
| Justification | Evidence suggested that continuing training programmes may improve the skills of healthcare professionals in delivering IO services and therefore increase service quality.[10, 11] |
| **Domain** | **Inviting Chinese Mainland TCM experts to set up training sites** |
| Action(s) | Under IMCGC’s supervision, invites TCM experts from Mainland China to provide insights into establishing professional training sites and demonstrate the IO service delivery process to all healthcare professionals, regardless of their involvement in IO. This is supposed to improve their practical skills, motivation, and confidence in delivering IO services. |
| Target(s) of the action | All healthcare professionals |
|  | Expertise of healthcare professionals |
| Temporality | Continuous |
| Implementation outcome(s) affected | Fidelity; sustainability |
| Justification | Evidence suggested that simulation-based education may have a higher learning effectiveness compared to non-simulation-based education.[22] |
| **Domain** | **Inviting patients and caregivers to share their experience using IO services** |
| Action(s) | Organises workshops and other campaigns to invite patients and caregivers who utilised IO services to share their first-hand experience, allowing all healthcare professionals, regardless of their involvement in IO, to have an emotional understanding on the value of the service from the users’ perspective. |
| Target(s) of the action | All healthcare professionals |
|  | Healthcare professionals’ thoughts about patients and caregivers endorsing IO |
| Temporality | Continuous |
| Implementation outcome(s) affected | Acceptability; adoption |
| Justification | Evidence suggested that patients’ and caregivers’ feedback may motivate healthcare professionals’ involvement in IO.[17] |
| **Domain** | **Explaining the fees of IO services and relevant subsidy schemes available to patients and caregivers** |
| Action(s) | Disseminate information regarding the fees of, and subsidy schemes for, IO services via different types of media (e.g., traditional and social media) |
| Target(s) of the action | Patients and caregivers |
|  | Knowledge about the fees of, and subsidy schemes for, IO services in Hong Kong and how they can be subsidised |
| Temporality | Continuous |
| Implementation outcome(s) affected | Adoption; feasibility |
| Justification | Evidence suggested that people receiving the cash transfers regarded such financial support as helpful in the short term, and sometimes in the long term. The cash allowed them to make more independent healthcare decisions.[2] |

BMD: Biomedically trained doctor; DHC: District Health Centre; IMCGC: Integrative Medicine Clinical Governance Centre; IO: Integrative oncology; TCM: Traditional Chinese medicine; TCMP: Traditional Chinese medicine practitioner.

**Table S4.** Implementation interventions to be delivered by the traditional Chinee medicine professional bodies

| **Domain** | **Elucidating TCM’s modern development to the general public** |
| --- | --- |
| Action(s) | Via different types of media (e.g., traditional and social media), presents and explains TCM’s modern development, including current clinical evidence supporting the effectiveness and safety of various TCM modalities, to the general public to boost their confidence in IO and TCM |
| Target(s) of the action | The general public |
|  | Knowledge about IO and recent TCM development |
| Temporality | Continuous |
| Implementation outcome(s) affected | Adoption; acceptability |
| Justification | Evidence suggested that patient decision aids (e.g., pamphlets or videos) may increase patients’ knowledge and expectations of benefits and harms of health treatments and screenings and, therefore, improve their participation in decision-making.[23] |
| **Domain** | **Promoting IO services to the general public** |
| Action(s) | Via different types of media (e.g., traditional and social media), presents and explains the safety and effectiveness of IO services and promotes IO as one of the evidence-based interventions for cancer |
| Target(s) of the action | The general public |
|  | Confidence in utilising IO services |
| Temporality | Continuous |
| Implementation outcome(s) affected | Adoption; acceptability |
| Justification | Evidence suggested that the availability of positive clinical outcomes (effectiveness and safety) is a major contributor to healthcare professionals’ and the public’s acceptance of IO services.[15] |
| **Domain** | **Organising campaigns to promote IO services as a key healthcare initiative endorsed by the government and the public** |
| Action(s) | Organises campaigns to raise healthcare professionals’ awareness of IO being one of the key healthcare interventions endorsed and supported by the Hong Kong Government and the public |
| Target(s) of the action | All healthcare professionals |
|  | Healthcare professionals’ awareness of IO as a government policy in response to high public demand. |
| Temporality | Continuous |
| Implementation outcome(s) affected | Adoption; acceptability |
| Justification | Evidence suggested that branding the delivery of integrative medicine as a way to respond to patients’ demands for holistic care may be useful to draw the attention of healthcare professionals.[17] |

IO: Integrative oncology; TCM: Traditional Chinese medicine.

**References**

1 Lim E, Vardy JL, Oh B, Dhillon HM. Mixed Method Study to Investigate Models of Australian Integrative Oncology. *J Altern Complement Med*. 2017;**23**(12):980-8.

2 Yoshino CA, Sidney-Annerstedt K, Wingfield T, Kirubi B, Viney K, Boccia D, et al. Experiences of conditional and unconditional cash transfers intended for improving health outcomes and health service usea qualitative evidence synthesis. *Cochrane Database Syst Rev*. 2023;**3**(3):Cd013635.

3 Lin V, Canaway R, Carter B. Interface, interaction and integrationhow people with chronic disease in Australia manage CAM and conventional medical services. *Health Expect*. 2015;**18**(6):2651-65.

4 Freeman T. Using performance indicators to improve health care quality in the public sectora review of the literature. *Health Serv Manage Res*. 2002;**15**(2):126-37.

5 Gibbons C, Porter I, Gonçalves-Bradley DC, Stoilov S, Ricci-Cabello I, Tsangaris E, et al. Routine provision of feedback from patient-reported outcome measurements to healthcare providers and patients in clinical practice. *Cochrane Database Syst Rev*. 2021;**10**(10):Cd011589.

6 Matus J, Walker A, Mickan S. Research capacity building frameworks for allied health professionals - a systematic review. *BMC Health Serv Res*. 2018;**18**(1):716.

7 Dudley L, Garner P. Strategies for integrating primary health services in low- and middle-income countries at the point of delivery. *Cochrane Database Syst Rev*. 2011;**2011**(7):Cd003318.

8 Boon HS, Kachan N. Integrative medicinea tale of two clinics. *BMC Complement Altern Med*. 2008;**:**32.

9 Witt CM. Training Oncology Physicians to Advise Their Patients on Complementary and Integrative Medicine. *J Altern Complement Med*. 2018;**24**(9-10):1016-7.

10 Ben-Arye E, Paller CJ, Lopez AM, White S, Pendleton E, Kienle GS, et al. The Society for Integrative Oncology Practice Recommendations for online consultation and treatment during the COVID-19 pandemic. *Support Care Cancer*. 2021;**29**(10):6155-65.

11 Ben-Arye E, Portalupi E, Keshet Y, Bonucci M, Can G, Kading Y, et al. Enhancing Palliative Care With Mindful TouchImpact of a Manual and Movement Therapy Training Program in an International Multidisciplinary Integrative Oncology Setting. *J Pain Symptom Manage*. 2021;**61**(2):229-36.

12 Victoor A, Delnoij DM, Friele RD, Rademakers JJ. Determinants of patient choice of healthcare providersa scoping review. *BMC Health Serv Res*. 2012;**:**272.

13 Hollenberg D. Uncharted groundpatterns of professional interaction among complementary/alternative and biomedical practitioners in integrative health care settings. *Soc Sci Med*. 2006;**62**(3):731-44.

14 D'Amour D, Goulet L, Labadie JF, Martín-Rodriguez LS, Pineault R. A model and typology of collaboration between professionals in healthcare organizations. *BMC Health Serv Res*. 2008;**:**188.

15 Kweku Sey A, Hunter J. Finding the value in oncology massageA mixed-method study of cancer services and survivors in Australia. *Adv Integr Med*. 2020;**7**(3):126-34.

16 Shojania KG, Jennings A, Mayhew A, Ramsay CR, Eccles MP, Grimshaw J. The effects of on-screen, point of care computer reminders on processes and outcomes of care. *Cochrane Database Syst Rev*. 2009;**2009**(3):Cd001096.

17 Chung VCH, Ho L, Leung TH, Wong CHL. Designing delivery models of traditional and complementary medicine servicesa review of international experiences. *Br Med Bull*. 2021;**137**(1):70-81.

18 Mittring N, Pérard M, Witt CM. Corporate culture assessments in integrative oncologya qualitative case study of two integrative oncology centers. *Evid Based Complement Alternat Med*. 2013;**20:**316950.

19 Rutert B, Stritter W, Eggert A, Auge U, Laengler A, Seifert G, et al. Development of an Integrative Care Program in a Pediatric Oncology Unit. *Complement Med Res*. 2021;**28**(2):131-8.

20 Wye L, Shaw A, Sharp D. Designing a 'NHS friendly' complementary therapy servicea qualitative case study. *BMC Health Serv Res*. 2008;**:**173.

21 Soklaridis S, Kelner M, Love RL, Cassidy JD. Integrative health care in a hospital settingcommunication patterns between CAM and biomedical practitioners. *J Interprof Care*. 2009;**23**(6):655-67.

22 Delisle M, Ward MAR, Pradarelli JC, Panda N, Howard JD, Hannenberg AA. Comparing the Learning Effectiveness of Healthcare Simulation in the Observer Versus Active RoleSystematic Review and Meta-Analysis. *Simul Healthc*. 2019;**14**(5):318-32.

23 Stacey D, Lewis KB, Smith M, Carley M, Volk R, Douglas EE, et al. Decision aids for people facing health treatment or screening decisions. *Cochrane Database Syst Rev*. 2024;**1**(1):Cd001431.
